# Neighborhood-Level Mass Incarceration and Future Preterm Birth Risk among African American Women

**DOI:** 10.1007/s11524-020-00426-w

**Published:** 2020-02-24

**Authors:** Shawnita Sealy-Jefferson, Brittney Butler, Townsand Price-Spratlen, Rhonda K. Dailey, Dawn P. Misra

**Affiliations:** 1 College of Public Health, Division of Epidemiology, Ohio State University, 1841 Neil Avenue, room 344, Columbus, OH 43210 USA; 2 College of Liberal Arts and Sciences, Department of Sociology, Ohio State University, Columbus, OH USA; 3 School of Medicine, Department of Family Medicine and Public Health Sciences, Wayne State University, Detroit, MI USA

**Keywords:** Mass incarceration, Preterm birth, Neighborhood, Urban health, Epidemiology, Public health, Minority health

## Abstract

While evidence for neighborhood effects on adverse birth outcomes is growing, no studies have examined whether living in a neighborhood impacted by mass incarceration is associated with preterm birth risk. We used modified Poisson regression to test whether residence in a neighborhood impacted by mass incarceration predicted future risk of preterm birth, among African American women. We linked data from the Justice Atlas of Sentencing and Corrections to survey and medical record data from the Life-course Influences on Fetal Environments study (*n* = 681). We also tested for effect modification by age and marital status. The association between prison admission expenditures and future risk of PTB varied by maternal age at birth, with younger women (< 35) having a modest increase in risk (relative risk (RR) 1.07; 95% confidence interval (CI) 0.99, 1.15), and older (35+ year old) women having lower risk (RR 0.86; 95% CI 0.69, 1.07). The association between the number of prison admissions due to new court cases and future risk of PTB varied by marital status, with evidence that married women may be protected (RR 0.75; 95% CI 0.61, 0.92), while little evidence of association was observed among unmarried women (RR 1.02; 95% CI 0.80, 1.30). The association between residence in an area impacted by mass incarceration and future risk of PTB among African American women may vary by age and marital status. Future research to identify the mechanisms of these associations is warranted.

## Introduction

**Fig. 1:**
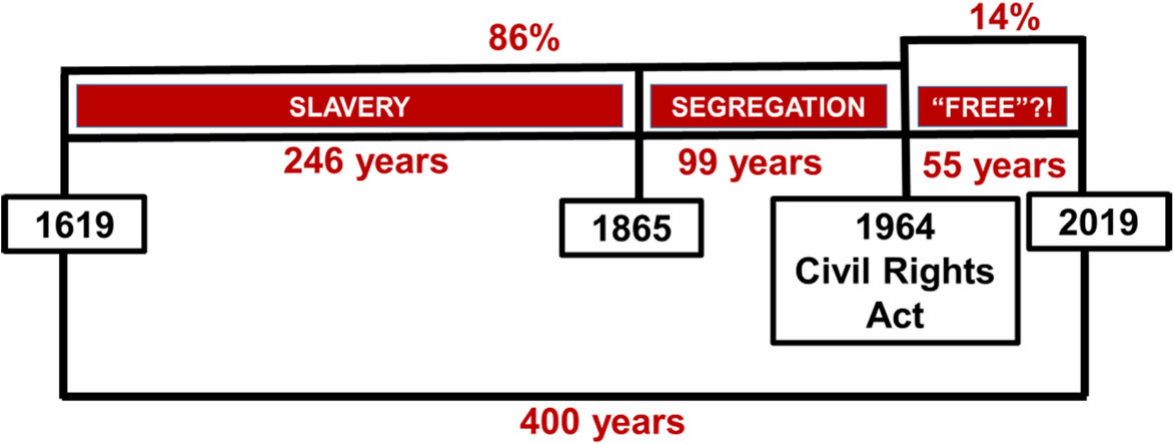
400-Year history of African Americans in the USA (1619–2019)

Preterm birth (PTB), or birth before 37 completed weeks of gestation, is an important public health issue [1], as it is the leading cause of infant mortality. Significant and seemingly intractable racial disparities in PTB exist, with African Americans disproportionately affected [2]. PTB is thought to result from the interaction of social, individual, and environmental factors, yet much of the literature on predictors of risk focuses on biologic and behavioral risk factors [3]. Importantly, known risk factors for PTB do not account for the increased risk among African Americans. In order to fully understand the disproportionate burden of poor health and mortality experienced by African Americans, an understanding of the unique 400-year history of this group, in the USA, is key (Fig. 1). Specifically, only 14% of the 400-year history of African Americans in this country is post Civil Rights Act of 1964.

Globally, the USA has the largest proportion of residents in jail or prison [4]. In the 31 years from 1978 to 2009, the number of prisoners held in federal and state prisons in the USA increased almost 430%, from 294,400 to 1,555,600 people [5]. Approximately 50% of African American women have an imprisoned relative, compared with 12% of their white counterparts [6]. Mass incarceration is a dominant social determinant of health in urban communities because it punishes accused offenders and appears to be a fundamental cause of persistent inequality [7]. Many urban areas have been deemed “Million Dollar Blocks” because at least one million taxpayer dollars are spent annually incarcerating residents of a single city block [8].

Neighborhoods with elevated incarceration rates also have poor population health (i.e., asthma, sexually transmitted infections, and psychiatric morbidity) [9–11], and increased crime rates [12]. Seminal studies have examined the impact of individual and family member incarceration and generally find positive associations with adverse birth outcomes [13–15]. In a state-level analysis, the imprisonment rate was a significant predictor of the total infant mortality rate, the Black infant mortality rate, and the racial disparity in infant mortality rates [15]. However, few studies have examined the contextual effect of neighborhood mass incarceration on individual risk of PTB. Furthermore, while there is a rapidly growing body of research on the importance of the preconception period for risk of adverse birth outcomes [16, 17], none thus far has examined the potential role of neighborhood incarceration rates in the period preceding a woman’s pregnancy on risk of PTB.

To fill this gap in the literature, we drew from a modified social ecological model [18] and an integrated perinatal health framework [16], and used data from the Life-course Influences on Fetal Environments (LIFE) study to examine whether preconception (up to 2 years prior to birth) neighborhood mass incarceration is positively associated with future risk of PTB among African American women. We also examined effect modification by (1) maternal age at birth, to test the weathering hypothesis; and (2) marital status given literature on the protective effects of marriage on PTB risk.

## Methods

### Study Population

Details of the LIFE study have been published [19]. Briefly, LIFE is a retrospective cohort, with enrollment occurring from 2009 to 2011. Self-identified African American women (≥ 18 years old) from Metropolitan Detroit Michigan who delivered a singleton infant were recruited from a suburban hospital. Women were excluded from the LIFE study if they (1) did not speak English; or (2) were mentally impaired, had serious cognitive deficits, or significant mental illness on the basis of history or any prior records. The final sample included 1410 women, which represented 71% of the women approached for study participation. Study participants completed detailed in-person interviews during the postpartum hospital stay and we abstracted their medical history from medical records. To quantify the impact of preconception neighborhood mass incarceration on future risk of PTB, we created a subset of the original cohort which included women who enrolled in 2010 and reported their address 2 years before enrolling in the study (*n* = 493), and those who enrolled in 2009 and reported living at their current residence for at least 12 months (*n* = 189). Our analytic sample included data from 48% of the original cohort (*n* = 682). The LIFE study was approved by the institutional review boards at the University of Michigan, St. John Providence Health Systems, and Wayne State University. All study participants gave written informed consent.

### Exposures

We spatially linked zip code–level data on prison admissions, releases, and paroles from publicly available data from the Justice Atlas of Sentencing and Corrections (which only reports data at the zip code areal unit) for the year 2008 to the subset of the LIFE study who reported their zip code for the same year. Prison admission, parole, and release rates were defined per 1000 adults. We also used measures of the type of prison admission including percent of people admitted to prison on the basis of a new conviction through the court, and the percent of those who were admitted on the basis of a revocation from parole or probation supervision. We also examined measures of the prevalence of admissions, measured as percent of the number of people admitted to prison, and percent of the total number of residents admitted to prison. Finally, we used a measure of the estimated cost of imprisonment for people admitted to prison, calculated by multiplying the average per day cost of imprisonment by the total estimated length of stay in prisons for all the people included in the geographic area.

### Outcome

We defined PTB as birth before 37 completed weeks of gestation, as ascertained from medical records. We used a hierarchical algorithm to categorize gestational age, with priority given to the estimate based on early ultrasound (between 6 and 20 weeks of gestation). In our analysis subsample, births occurred in the years 2009 and 2010.

### Statistical Analysis

We used univariate and bivariate statistics to describe the data, with chi-square tests and Wilcoxon rank sum tests for categorical and continuous variables, respectively. We used Pearson correlations to estimate the relationship between the mass incarceration variables. We assessed all variables for missing data (range of missingness across all variables was between 0 and 7%), and we used list-wise deletion in statistical models [20]. Since the prevalence of our outcome was > 10%, and there was small block-group level variation in PTB (precluding hierarchical modeling (ICC = 5.7%)) [21], we estimated modified Poisson regression models with robust error variance [22], which approximated relative risks (RR) and associated 95% confidence intervals (95% CI) for the association between each mass incarceration variable (separately) and risk of PTB. The mass incarceration variables were modeled continuously, and were rescaled by their interquartile ranges, to allow us to interpret the results as the risk of PTB in women who resided in neighborhoods with high (75th percentile of the distribution) versus low (25th percentile of the distribution) measures of mass incarceration. We included separate interaction terms in our regression models to test heterogeneity of the associations by self-reported maternal age (< 35 and 35+ years, given that age 35+ years is a known risk factor for PTB) and marital status (yes/no), and discuss stratum specific results (if *p* values for the interaction terms were < 0.10). We heed recent calls to abandon reliance on statistical significance in interpreting research results, in favor of more detailed and nuanced presentation and of statistical analyses, with the recognition that *p* values and decisions about what research ideas should be explored further have no association [23]. Given the literature on neighborhood effects on PTB among African American women which identified few true confounders [19, 24], and the literature on the community effects of mass incarceration [14], sociodemographic variables may lie on the pathway linking preconception neighborhood mass incarceration to PTB. Adjusting for potential mediators would compromise the precision of our estimates, and attenuate the associations we were trying to identify [25]; we therefore present unadjusted models. We used SAS version 9.4 for Windows (SAS Institute Inc., Cary, NC) for all analyses.

## Results

The mean age of the study participants was 27 years: 16.3% had a PTB (*n* = 111) (Table 1). Over 70% of the sample were unmarried, almost three quarters had at least 12 years of education, and 49 % of the sample had an annual income of $35,000 or more. Nearly 50% reported depressive symptoms above the median for the analysis subset. Of the women who were 18–34 years old, 16.1% had a PTB and 23% were married at the time of their birth, while 17.4% of women 35+ years old had a PTB and 50% were married at the time of their birth (data not shown).

**Table 1:** Demographic and psychosocial factors of the Life-course Influences on Fetal Environments study participants (2009–2011)

	Total sample (*n* = 681) *N* (%)	Term delivery (*n* = 570) *N* (%)	Preterm (*n* = 111) *N* (%)	RR	95% CI
Age					
18–19	56 (8.22)	45 (7.89)	11 (9.91)	1.26	0.68, 2.36
20–24	206 (30.25)	171 (30.00)	35 (31.53)	1.09	0.70, 1.71
25–29	193 (28.34)	163 (28.60)	30 (27.03)	*Referent*	
30–34	128 (18.80)	110 (19.30)	18 (16.22)	0.90	0.53, 1.55
35+	98 (14.39)	81 (14.21)	17 (15.32)	1.10	0.64, 1.90
Married					
No	490 (72.8)	409 (72.65)	81 (73.64)	*Referent*	
Yes	183 (27.19)	154 (27.35)	29 (26.36)	0.96	0.65, 1.41
Education (years)					
≤ 12	183 (26.87)	155 (27.19)	28 (25.23)	*Referent*	
> 12	498 (73.13)	415 (72.81)	83 (74.77)	1.09	0.74, 1.61
Income					
Under $35,000	299 (47.31)	254 (48.02)	45 (43.69)	1.16	0.81, 1.65
$35,000 or more	333 (52.69)	275 (51.98)	58 (56.31)	*Referent*	
Depressive symptoms (median split)					
< 9	300 (47.39)	253 (47.56)	47 (46.53)	*Referent*	
≥ 9	333 (52.61)	279 (52.44)	54 (53.47)	1.04	0.72, 1.48

Study participants resided in *n* = 99 zip codes, with between 1 and 59 women per zip code. Overall, for 2008, the state of Michigan had records on 9713 prison admissions, 13,414 prison releases, and 20,380 paroles. Of these, 95% of admissions, 93.9% of releases, and 65.6% of paroles were mapped to in-state zip codes. Statewide, Michigan had a prison admission rate of 1.45, a release rate of 1.99, and a parole rate of 2.13/1000 residents. Average estimated zip code–level expenditures for prison admissions, across all zip codes in the state, was $1.2 million.

In our sample, average prison admission costs were $9.43 million, nearly tenfold higher than the overall zip code average in the state (Table 2). Approximately 52% of the admissions in these neighborhoods were due to revoked parole and probation compared with 48% from new court cases. On average, 2% of the total number of adult residents per zip code were admitted to prison. Average release and parole rates were 3.65 and 3.67 per 1000 adults, respectively. The strongest correlation was between the prison release and parole rates (*ρ* = 0.98).

**Table 2:** Correlations between preconception zip code–level mass incarceration variables: Life-course Influences on Fetal Environments study (*n* = 681; 2009–2011)

Variable no.		1	2	3	4	5	6	7	8
1	Admission rate/1000	1							
2	Admission cost (millions of dollars)	0.86	1						
3	Percent revoked parole/probation	− 0.17	− 0.22	1					
4	Percent new court cases	0.22	0.26	− 0.83	1				
5	Percent of total number of adult residents admitted	0.02	0.31	− 0.19	0.24	1			
6	Percent of total number of people admitted to prison	0.67	0.62	− 0.18	0.23	0.52	1		
7	Release rate/1000	0.74	0.56	− 0.04	0.08	− 0.12	0.38	1	
8	Parole rate/1000	0.69	0.48	− 0.01	0.05	− 0.17	0.32	0.98	1
	Mean	2.81	9.43	51.54	48.14	2.12	3.09	3.65	3.67
	Median	2.82	10.60	50.50	49.50	2.10	2.80	4.17	3.75
	Standard deviation	1.80	7.37	9.57	9.52	0.71	1.93	2.79	3.05
	Minimum	0	0	0	0	0	0	0	0
	Maximum	10.56	29	100	80	5.5	16.1	23.12	26.68

In the overall sample, we observed little evidence of an association between any of the preconception zip code–level mass incarceration measures and future risk of PTB (Table 3). However, we observed evidence suggestive of effect modification by maternal age at birth for neighborhood-level costs associated with imprisonment (*p* = 0.07), and by marital status for prison admission stemming from new court cases (*p* = 0.06). Among women aged 18–34 (*n* = 583), those who lived in a neighborhood with high compared with low prison admission costs had increased risk of having a PTB (RR 1.07; 95% CI 0.99, 1.15). However, for women 35+ (*n* = 98), the associations were less precise and in the opposite direction (RR 0.86; 95% CI 0.69, 1.07). Next, women who were married at the time of birth (*n* = 183) and lived in neighborhoods with high versus low prison admissions due to new court cases had lower risk of PTB (RR 0.75; 95% CI 0.61, 0.92). There was little evidence of an association between preconception zip code prison admissions due to new court cases and PTB, among unmarried women (*n* = 490; RR 1.02; 95% CI 0.80, 1.30).

**Table 3:** Associations between preconception mass incarceration and risk of preterm birth in the total sample and results stratified by age and marital status for joint associations: Life-course Influences on Fetal Environments Study (2009–2011)

Preconception zip code mass incarceration variables (75th vs. 25th percentile)	Total sample (*n* = 681) Relative risk (95% confidence interval)	Age		Married	
		< 35 years (*n* = 583)	35+ years (*n* = 98)	Yes (*n* = 183)	No (*n* = 490)
Prison admission rate/1000	1.24 (0.90, 1.72)				
Prison admission cost (millions of dollars)	1.04 (0.97, 1.12)	1.07 (0.99, 1.15)	0.86 (0.69, 1.07)		
Percent of admissions due to revoked probation/parole	1.02 (0.82, 1.27)				
Percent of admissions due to new court cases	0.92 (0.77, 1.09)			0.75 (0.61, 0.92)	1.02 (0.80, 1.30)
Percent of the total number of adult residents admitted	1.02 (0.86, 1.23)				
Percent of the total number of people admitted to prison	0.96 (0.76, 1.23)				
Prison release rate/1000	1.04 (0.96, 1.12)				
Parole rate/1000	1.05 (0.91, 1.21)				

Age × prison admission cost interaction *p* value: 0.07

Marital status × prison admissions due to new court cases interaction *p* value: 0.06

## Discussion

We found that higher preconception zip code prison admission costs predicted higher PTB risk among women who were < 35 years old, and a less precise yet opposite effect among older women. We also observed apparent buffering of the impact of zip code prison admissions due to new court cases on PTB risk among married women, but not unmarried women. Lastly, we document that our study subsample lived in zip codes that had, on average, expended approximately $9.4 million in 2008 on incarcerating individuals, compared with an average across zip codes in the entire state of Michigan, which expended $1.2 million.

Two existing state-level analyses revealed positive associations between imprisonment and infant mortality rates [14, 15]. One existing study using Pregnancy Risk Assessment Monitoring System data from 1990 to 2003 found that maternal and/or paternal incarceration was a significant predictor of infant mortality [15]. Our work extends this literature because we examine the impact of preconception zip code–level mass incarceration on individual risk of PTB, among African American women. Our work suggests that the removal of persons from communities through prison admission and the associated estimated expenditures may have collateral consequences, including age- and marital status–dependent effects on risk of adverse birth outcomes. Chronic stress may be one mechanism by which neighborhood-level mass incarceration may affect future PTB risk for African American women living in these areas. The threat of arrest in these areas may contribute to chronic stress which is a known trigger for the inflammatory response system, which has been implicated in the etiology of PTB [26].

The reason for the apparent heterogeneity of the association between preconception prison expenditures and risk of PTB by maternal age at birth is unclear. However, money spent by states on corrections may decrease the investment for social program spending [27], which provides population health benefits [28]. Specifically, every $1.00 increase in state corrections spending is associated with a $1.40 decrease in social program spending [28, 29]. Given that younger mothers are more likely to utilize these social services [30], underfunding impacts the range and availability of services in these communities. Older women may also have more social capital to buffer against the influences of living in such residential areas. More research to confirm and understand this association is warranted.

The “revolving door” of prison admissions is largely caused by the return of previously incarcerated individuals to prison [31–33]. These prison returns occur as a result of new accused crimes and technical violations of community supervision (or parole) [34]. Since prior imprisonment is stigmatizing, the risk of being sentenced to prison after a new court case may be higher for those on community supervision or with a prior record of incarceration [34]. Our finding is that women who lived in zip codes with high (versus low) prison admissions due to new court cases during the preconception period, but who were married at the time of birth, were protected from PTB. The pathways through which this protection might operate among married women include increased financial security, access to healthcare, and social support. Among unmarried women, we observed little evidence of an association between this measure of preconception prison admission and risk of PTB. In our sample, older women were more likely to be married than younger women. Indeed, research has shown that marital status is most protective against PTB among older mothers (> 34 years). In our study, we do not have data on the duration of marriage, which could give us clues about this relationship over time. Future longitudinal research is needed to understand the mechanism of this association, and to test a 3-way interaction between maternal age, marital status, and neighborhood prison admission measures.

The collateral damage on African American communities caused by mass incarceration has been in the past and continues to be substantial. In some states, the right to vote is limited to individuals without felony convictions [35], which ensures limited political power even for returning citizens. Recent estimates suggest that 13.2% of African Americans have been disenfranchised [36]. Political disenfranchisement could help to ensure that conservatives win political elections [37], which could reduce the distribution of resources allotted to these communities, and exacerbate or solidify inequities in health outcomes, including infant mortality among African Americans [27, 38]. Neighborhoods impacted by mass incarceration also have lower community-level social control [39], which results in decreased neighborhood safety [40]. These neighborhoods also have an overrepresentation of alternative financial service providers including check-cashers, payday lenders, and pawnshops, which intentionally extract financial resources from these communities [29].

## Limitations

The results of the current study should be interpreted in light of the following limitations. First, the study participant or their immediate family members’ criminal justice system contact was unmeasured and may confound the associations reported here. Second, the Justice Atlas of Sentencing and Corrections used zip codes as a surrogate for neighborhoods. Multiple cities and neighborhoods can have the same zip code, and it is likely that average rates in a particular neighborhood could vary more in zip codes than census tracts or block groups [11, 41]. Next, the prison admissions expenditure variable is an estimate that may not accurately capture the exact duration of time spent in prison, especially if prisoners serve a percentage less than 100% of their sentence. Further, the consistency, accuracy, and completeness of home residence address data for people entering prison are self-reported and not verified by independent sources. However, it is unclear how misreporting address information would bias our estimates. Next, the range of missing data in our analytic sample ranged from 0 to 7% (which we assumed occured at random), and was not large enough to justify imputation. Finally, the LIFE study sample was recruited from one suburban hospital, which could limit the generalizability of our findings. However, the overall LIFE sample had similar sociodemographic characteristics and birth outcomes as non-Hispanic Black and African American women in the USA, the State of Michigan, and Wayne County, MI. In addition, the study recruitment hospital was chosen for its wide catchment area, the heterogeneity of patients (from 64 municipalities and 3 counties), and the large number of births per year. Given the novelty of our data, and the hypothesis-generating nature of our research questions, we did not adjust for multiple comparisons.

## Strengths

Our study has several strengths that add to and extend the existing neighborhood effects on preterm birth literature. First, we answer for the first time: does living in a neighborhood impacted by mass incarceration before conception increase future risk for preterm birth? And if so, are there buffering or exacerbating factors? We linked study participant residential zip codes from 2008 to publicly available data on mass incarcerations from 2008, and examined the impact of eight measures of mass incarceration on risk of PTB. We also leveraged an entirely African American cohort, which allowed us to test for effect modification within this high-risk group. Mass incarceration is both a reflector and a participator of structuring racial health inequities in the USA [42], and our methodology extends the existing literature on the collateral damage of mass incarceration on a historically marginalized segment of our population. Next, our study participants were recruited in the immediate postpartum period, which increased the heterogeneity of risk of PTB in our cohort (because our study includes women with complete, incomplete, and no prenatal care), compared with studies which only include women who receive complete prenatal care (and thus have lower PTB risk).

## Summary

We report suggestive evidence that residence in neighborhoods with high prison admission costs before pregnancy may be associated with higher future risk of PTB among younger African American women. Our results also suggest that marital status at the time of birth might protect against future risk of PTB among those living in neighborhoods with high prison admissions due to new court cases, during the preconception period. Mass incarceration is an important macrosocial determinant of health inequities, and should be the focus of future quantitative and qualitative research.
